# New Data on the Features of Skin Barrier in Hidradenitis Suppurativa

**DOI:** 10.3390/biomedicines11010127

**Published:** 2023-01-04

**Authors:** Orsolya Somogyi, Zsolt Dajnoki, Lilla Szabó, Krisztián Gáspár, Zoltán Hendrik, Christos C. Zouboulis, Klaudia Dócs, Péter Szücs, Katalin Dull, Dániel Törőcsik, Anikó Kapitány, Andrea Szegedi

**Affiliations:** 1 Department of Dermatology, MTA Centre of Excellence, Faculty of Medicine, University of Debrecen, H-4032 Debrecen, Hungary; 2 ELKH-DE Allergology Research Group, H-4032 Debrecen, Hungary; 3 Petrányi Gyula Doctoral School, University of Debrecen, H-4032 Debrecen, Hungary; 4 Department of Pathology, Faculty of Medicine, University of Debrecen, H-4032 Debrecen, Hungary; 5 Departments of Dermatology, Venereology, Allergology and Immunology, Dessau Medical Center, Brandenburg Medical School Theodor Fontane and Faculty of Health Sciences Brandenburg, D-06847 Dessau, Germany; 6 Department of Anatomy, Histology and Embryology, Faculty of Medicine, University of Debrecen, H-4032 Debrecen, Hungary

**Keywords:** hidradenitis suppurativa, permeability barrier, desmosome, tight junction, keratinocyte

## Abstract

Hidradenitis suppurativa (HS) is a Th1/17-driven inflammatory skin disease of the apocrine gland-rich (AGR) skin regions, where keratinocytes seem to be the crucial drivers of the initial pathogenic steps. However, the possible role of permeability barrier alteration in activating keratinocytes during HS development has not been clarified. We compared the major permeability barrier elements of non-lesional HS (HS-NL; n = 10) and lesional HS (HS-L; n = 10) skin with healthy AGR regions (n = 10) via RT-qPCR and immunohistochemistry. Stratum corneum components related to cornified envelope formation, corneocyte desquamation and (corneo)desmosome organization were analyzed along with tight junction molecules and barrier alarmins. The permeability barrier function was also investigated with transepidermal water loss (TEWL) measurements (n = 16). Junction structures were also visualized using confocal microscopy. At the gene level, none of the investigated molecules were significantly altered in HS-NL skin, while 11 molecules changed significantly in HS-L skin versus control. At the protein level, the investigated molecules were similarly expressed in HS-NL and AGR skin. In HS-L skin, only slight changes were detected; however, differences did not show a unidirectional alteration, as KRT1 and KLK5 were detected in decreased levels, and KLK7, KRT6 and DSG1 in increased levels. No significant differences in TEWL or the expression of junction structures were assessed. Our findings suggest that the permeability barrier is not significantly damaged in HS skin and permeability barrier alterations are not the driver factors of keratinocyte activation in this disease.

## 1. Introduction

Hidradenitis suppurativa (HS) is a recurring chronic inflammatory skin disease primarily affecting hair follicles in apocrine gland-rich (AGR) skin regions. The estimated prevalence of HS, which severely impairs the quality of life of affected individuals, is approximately 0.4% [1]. HS has multifactorial origins, including both genetic (dysregulation of the γ-secretase/Notch pathway) and environmental factors (lifestyle, hormonal status, immune and microbiota dysregulation) [2,3]. Although the pathogenesis of the disease is not fully understood, it has been revealed that HS is a Th1/Th17-mediated disease characterized by aberrant adaptive and innate immune reactions with marked increase in proinflammatory cytokines (IL-1, IL-17, IL-23 and TNF-α), T and B lymphocytes, plasma cells and dendritic cells [2]. Recently, the pivotal role of keratinocytes (KCs) in the development of this immune-mediated inflammation has emerged [4,5]. Moreover, it has been demonstrated that KCs play an important role even in the initiation of HS, as KCs in non-lesional HS skin produce not only antimicrobial peptides and IL-1β but also TNF-α and IL-23, supporting the driving role of KCs in the pathogenesis of HS [4,5]. However, it remains unclear how the activation of KCs is triggered. In addition to the endogenous γ-secretase mutations present in 6% of HS cases, the main exogenous factors associated with KC activation can be altered microbiota, skin temperature, moisture, pH or barrier damage. Therefore, we aimed to determine whether barrier alteration is present in HS.

In this study, the main permeability barrier elements, including the stratum corneum (SC) and tight junctions (TJs), were investigated at the mRNA and protein levels in skin biopsies from lesional (HS-L) and non-lesional (HS-NL) skin areas of HS patients and AGR skin from healthy individuals. Among the SC components, we examined molecules involved in the cornified envelope (CE) formation, corneocyte desquamation and desmosome formation in parallel with TJ elements and barrier alarmins. Moreover, the function of the permeability barrier and the organization of TJ and (corneo)desmosome elements were investigated using transepidermal water loss (TEWL) measurements and confocal microscopy.

Although differentially expressed barrier genes were identified, these differences could not be confirmed at the protein level. Moreover, no significant differences in the TEWL or in the microscopical detection of junction structure molecules were found among the three sample groups. Based on these results, the permeability barrier is not significantly damaged in HS skin, and barrier alterations are not likely to be the driver factors of KC activation in this disease.

## 2. Materials and Methods

### 2.1. Patients

HS patients from our outpatient clinic and healthy individuals (with no skin disease) were recruited in this study. HS patients were involved in the study based on the following inclusion criteria: individuals over 18 years with clinically diagnosed moderate-to-severe HS (based on Sartorius score), with disease duration of at least 6 months, biological therapy naïvety and conventional systemic therapies were washed out for 4 weeks, while topical treatments were terminated 4 days before skin biopsy and barrier measurements. Exclusion criteria: patients under therapy or/and a lack of written consent. Informed consent, according to the Declaration of Helsinki principles, was obtained from all patients before sampling. The study was approved by the Hungarian Medical Research Council and local ethics committee of the University of Debrecen, Hungary.

### 2.2. Skin Biopsies

Skin biopsies were collected from lesional and non-lesional (perilesional area, ≥3 cm from the lesion, which is thought to display a predisease phenotype) skin of 10 patients with HS and from the normal skin of 10 healthy individuals (samples from the axillary region representing AGR) (see age, sex and localization in Table S1). Using region-matched healthy control is essential, as distinct skin regions bear significantly different immune activity [6,7,8,9]. Part of each biopsy was stored in RNAlater (Qiagen, Hilden, Germany) at −70 °C until RNA isolation for RT-PCR, and another part of each biopsy was formalin-fixed and paraffin-embedded for immunohistochemistry (IHC). After hematoxylin and eosin (H&E) staining, samples were classified according to the number of apocrine glands and were defined as AGR (apocrine gland-rich) skin if n ≥ 2 apocrine glands were identified in the field of view at 100× magnification.

### 2.3. RNA Isolation, Reverse Transcription and Real-Time Quantitative PCR

All samples were homogenized in TriReagent solution (Sigma-Aldrich, Dorset, UK) with Tissue Lyser (QIAGEN) using innuSPEED Lysis Tubes prefilled with metal beads (Analytik Jena). Total RNA was isolated from skin biopsies. The concentrations and purities of the RNA samples were measured using a NanoDrop spectrophotometer (Thermo Scientific, Bioscience, Budapest, Hungary). RNA quality was checked using an Agilent 2100 Bioanalyser (Agilent, Santa Clara, CA, USA). RNA was reverse transcribed into complementary DNA (cDNA) using a High Capacity cDNA Archive Kit (Invitrogen, Life Technologies, San Francisco, CA, USA), according to the manufacturer’s instructions and the indicated thermal protocol. After treatment with DNase I (Applied Biosystems, Foster City, CA, USA), qRT-PCR reactions were performed in triplicate using pre-designed FAM-MGB assays and TaqMan^®^ Gene Expression Master Mix from Applied Biosystems (Life Technologies). The following oligo sets were used: PPIA (Hs99999904_m1), FLG (Hs00856927_g1), KRT1 (Hs00196158_m1), KRT10 (Hs00166289_m1), LOR (Hs01894962_s1), CLDN1 (Hs00221623_m1), CLDN23 (Hs01013638_s1), OCLN (Hs00170162_m1), CDH1 (Hs01023895_m1), DSC1 (Hs00245189_m1), CDSN (Hs00169911_m1), DSG1 (Hs00355084_m1), TGM5 (Hs00909973_m1), KLK5 (Hs01548153_m1), KLK7 (Hs00192503_m1), KLK14 (Hs00983577_m1), PKP1 (Hs00240873_m1), KRT6A (Hs01699178_g1), KRT16 (Hs00373910_g1). Reactions were performed on a LightCycler^®^ 480 System (Roche, Grenzach-Wyhlen, Germany). Relative mRNA levels were calculated using either the comparative Ct method or based on standard curves and normalized to the expression of PPIA mRNA.

### 2.4. Immunohistochemistry Staining and Evaluation

For IHC analyses, paraffin-embedded sections from HS patients and healthy control skin samples were deparaffinized and rehydrated. Endogenous peroxidase activity was eliminated by incubating sections in 3% H_2_O_2_ for 15 min. After heat-induced antigen retrieval, sections were blocked with 1% bovine serum albumin (BSA). Sections were incubated overnight at 4 °C with the following primary antibodies: human CDSN (rabbit polyclonal IgG, HPA054184, Sigma-Aldrich), human CLDN1 (rabbit polyclonal IgG, ab15098, Abcam, Cambridge, UK), human DSG1 (rabbit polyclonal IgG, NBP1-84567, Novus Biologicals, Centennial, CO, USA), human FLG (mouse monoclonal IgG [ab17808], Abcam), human KLK5 (rabbit polyclonal IgG, ab7283, Abcam), human KLK7 (rabbit, polyclonal IgG, NBP1-31428 Novus Biologicals), human KRT1 (rabbit monoclonal IgG, ab185628, Abcam), human KRT6 (mouse monoclonal IgG, ab18586, Abcam), human LOR (rabbit monoclonal IgG, NBP133610, Novus Biologicals), human OCLN (rabbit monoclonal IgG, ab216327, Abcam) and human TGM5 (rabbit polyclonal IgG, ab133786, Abcam). Subsequently, sections were incubated with an anti-mouse/rabbit (Dako) HRP-conjugated secondary antibody. Before and after incubating with antibodies, samples were washed with tris-buffered saline with 0.1% Tween for 5 min, 3 times each. Signals were detected with the Vector^®^ VIP and ImmPACT™ NovaRED™ Kit (VECTOR Laboratories, Burlingame, CA, USA). Background staining was performed with methylene green. The detection of each protein was carried out on all sections in parallel at the same time to evaluate comparable protein levels. Positive, IgG and isotype controls were also used to normalize staining against all proteins (mouse and rabbit IgG (Covalab), rabbit immunoglobulin fraction (Dako)). Skin specimens were also stained with H&E. Visual scoring of apocrine glands was performed by a professional pathologist. IHC sections were digitalized using a Zeiss Mirax Midi scanner (Zeiss, Oberkochen, Germany). Sections were evaluated using the HistoQuant application of the Pannoramic Viewer software (3DHistech, Budapest, Hungary). For each molecule, we trained the application separately on what constitutes a positive area (which pixel is positive) or background staining. At least 3 regions of interest (ROIs) were selected per slide with an epidermal area of 500 µm in length. The pre-trained algorithm was used to evaluate the ROIs in each section. Finally, the total staining intensity was determined and compared.

### 2.5. Transepidermal Water Loss Measurements

TEWL measurements were performed in HS patients (n = 16; 8 females, 8 males) and healthy individuals (n = 20; 9 females, 11 males) under standardized laboratory conditions at a temperature of 22–25 °C and a humidity level of 40–60%. Before the measurements, individuals were adapted to room conditions for 15 min. The Dermalab Combo (Cortex Technology, Hadsund, Denmark) was used to measure TEWL (g/hm^2^) on healthy axilla (representing the AGR area) and lesional and non-lesional areas of HS patients. Measurements were performed in triplicate for 30 s each.

### 2.6. Confocal Microscopy

For confocal microscopy, three 40 μm-thick skin paraffin-embedded specimens from HS-NL and healthy AGR sample groups were assessed, as previously described [10]. Imaging of the immunostained samples was carried out on an Olympus FV3000 confocal system using a 40× or 60× oil-immersion lens (NAs: 1.40–1.42, respectively). Acquisition settings (laser power, confocal aperture and gain, detector parameters) were identical for all samples. Series of 1 um thick optical sections with 0.5 µm separation in the Z axis were acquired. The overall number of optical sections for each sample was 6. No pixels corresponding to immunostained puncta were saturated. Images for the figures were processed using the Adobe Photoshop CS5 software.

Regularity of the CLDN and DSG1 immunostaining was analyzed by measuring the distance between two adjacent immunostained puncta along the cross section of the cell membrane (defined as a closed line around a DAPI stained nucleus). Inter-puncta distances (120–160 per condition) were measured in the samples. The distances were presented as box plots where the box range represents +/− SD, whiskers indicate the 1.5-fold IQR distance (Q3–Q1) outliers. All datapoints are plotted as grey dots. The mean value is shown as a hollow square within the box.

### 2.7. Statistical Analysis

The normality of data was tested by a Shapiro–Wilk test. One-way ANOVA and Tukey post hoc test (normal distribution) or Kruskal–Wallis test with Dunn’s post hoc test (not normal distribution) were used to determine statistical differences between the groups (* *p* < 0.05; ** *p* < 0.01; *** *p* < 0.001). Graphs show the median and the corresponding 95% upper and lower confidence intervals (boxes) and maximum and minimum values. Statistical analyses were performed using GraphPad Prism software version 7 (GraphPad Software Inc., San Diego, CA, USA).

## 3. Results

### 3.1. Gene Expression Profile of Barrier Molecules

To investigate the permeability barrier in HS at the molecular level, SC components, including elements of CE formation (structure proteins, FLG, LOR, KRT1, KRT10, TGM5), corneocyte desquamation (KLK5, KLK7, KLK14), desmosome formation (DSG1, DSC1, PKP1, CDSN), together with TJ proteins (CDH1, CLDN1, OCLN), and barrier alarmins (KRT6A, KRT16) were investigated at the gene level with RT-qPCR in lesional and non-lesional HS skin and in healthy skin samples as control (AGR).

**Figure 1:**
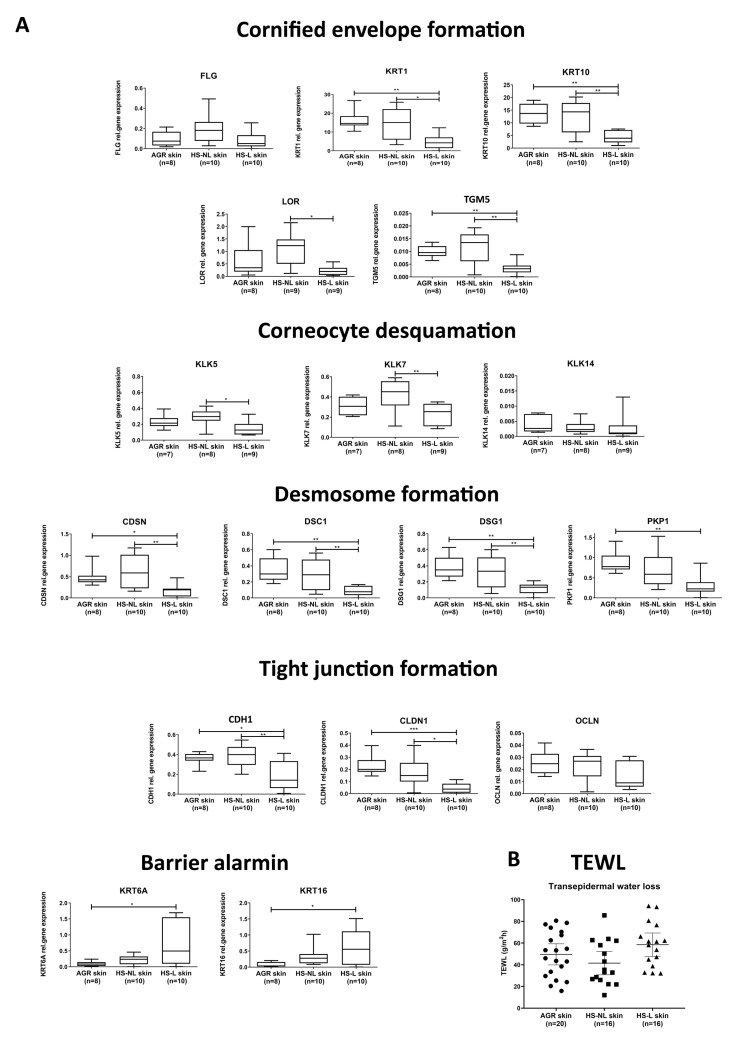
(**A**) Gene expression levels of permeability barrier components in healthy AGR, non-lesional HS and lesional HS skin examined with qRT-PCR. The graphs show the median ± 95% confidence interval of measured mRNA transcript levels (* *p* < 0.05; ** *p* < 0.01; *** *p* < 0.001, as determined by one-way analysis of variance followed by Sidak’s post hoc test in cases of normal distribution or Kruskal–Wallis test followed by Dunn’s post hoc test when data distribution was not normal). Abbreviations: AGR, apocrine gland rich; CDSN, corneodesmosin; CLDN, claudin; DSG1, desmoglein 1; DSC1, desmocollin; FLG, filaggrin; HS-L, HS lesional skin; HS-NL, HS non-lesional skin; KLK, kallikrein-related peptidase; KRT, keratin; LOR, loricrin; OCLN, occludin; PKP1, plakophilin; TGM, transglutaminase. (**B**) Transepidermal water loss levels in healthy AGR, non-lesional HS and lesional HS skin. Measurements were carried out with Dermalab Combo (Cortex Technology, Hadsund, Denmark) in the axillary regions of the involved healthy individuals and patients. Measurements were carried out in triplicate. Abbreviations: AGR, apocrine gland rich; HS-L, HS lesional skin; HS-NL, HS non-lesional skin.

No differences in SC and TJ components were detected between HS-NL and AGR samples (Figure 1 and Table S1). The expression levels of 11 molecules were significantly decreased in HS-L samples compared with HS-NL samples (Figure 1 and Table S2).

When comparing HS-L and AGR samples, the expression of 9 molecules was significantly decreased, while the expression of barrier alarmins KRT6 and KRT16 were significantly increased in HS-L specimens (Figure 1 and Table S2).

### 3.2. Protein Expression Profile of Barrier Molecules

Since gene expression levels do not always reflect the protein levels, which are the functional forms of the molecules, representatives of SC and TJ formation were investigated at the protein level with IHC. Following IHC, slides were digitalized by whole slide imaging, which allowed us to quantify protein levels with Pannoramic Viewer Software. It is important to emphasize that HS is a follicular disease; therefore, barrier molecules in HS-NL samples were analyzed separately in the interfollicular and the follicular epidermis. In HS-L samples, follicular epithelia could not be quantified due to the uneven distribution of hair follicles in the skin specimens.

**Figure 2:**
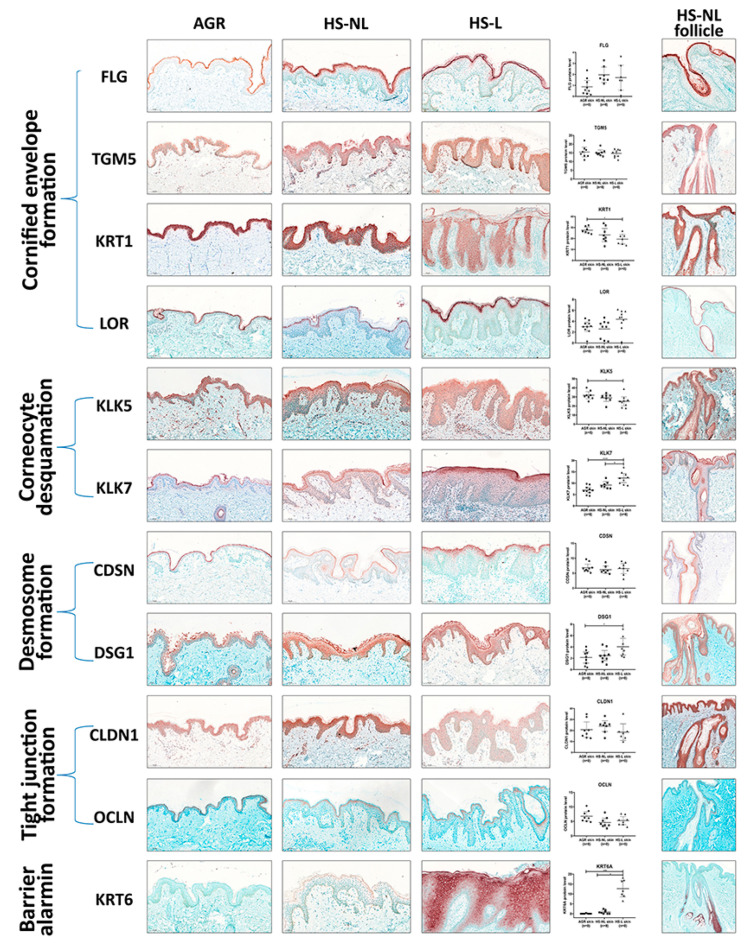
Representative images for immunostaining and epidermal quantification of SC and cell junction components in healthy AGR, non-lesional HS and lesional HS skin samples. Protein levels were blindly analyzed by Pannoramic Viewer software. In the last column, representative images for the follicular epidermal pattern of permeability barrier components in non-lesional HS skin samples were demonstrated. Size bars = 100 μm. The graphs show the median ±95% confidence interval of measured protein levels (* *p* < 0.05; ** *p* < 0.01; *** *p* < 0.001, as determined by one-way analysis of variance followed by Sidak’s post hoc test in case of normal distribution or Kruskal–Wallis test followed by Dunn’s post hoc test when data distribution was not normal). Abbreviations: AGR, apocrine gland rich; CDSN, corneodesmosin; CLDN, claudin; DSG1, desmoglein 1; FLG, filaggrin; HS, Hidradenitis suppurativa; HS-L, HS lesional skin; HS-NL, HS non-lesional skin; KLK, kallikrein-related peptidase; KRT, keratin; LOR, loricrin; OCLN, occludin; TGM, transglutaminase.

In HS-NL, when comparing the protein expression of barrier molecules in the interfollicular epidermis, the staining pattern of each molecule was similar to that of AGR samples. Although the amount of KRT6 was prominently but not significantly elevated in HS-NL samples (FC = 14.43), all other investigated molecules were similarly expressed between the two sample groups (Figure 2, Table S2).

The follicular epidermal staining pattern of each molecule was also similar in HS-NL and AGR samples. In HS-NL samples, the staining pattern did not show any differences between the interfollicular and follicular epidermis; in addition, barrier protein levels also seemed to be similar in the two epidermal regions (Figure 2).

In HS-L skin specimens, we could only examine and quantify the interfollicular epidermis at the protein level. In this epidermal part, the barrier alarmin KRT6 and the KLK7 enzyme, which takes part in corneocyte desquamation, was significantly upregulated in HS-L samples compared to KRT6 and KLK7 protein levels in the HS-NL and the AGR samples (Figure 2, Table S2). Among them, the KRT6 level was notably high in HS-L skin compared to both HS-NL and AGR skin (FC = 15.51 and 223.71, respectively), which was also reflected in its staining pattern. It was barely detectable in AGR skin, the SC stained positive for KRT6 in HS-NL skin samples, while the whole epidermis exhibited strong KRT6 positivity in HS-L samples. No differences in other SC and TJ molecules were detected at the protein level between HS-L and HS-NL samples.

When HS-L was compared to AGR samples, the results were somewhat ambiguous; besides KRT6 and KLK7, the desmosome component DSG1 was significantly upregulated while KRT1 and KLK5 were significantly downregulated (Figure 2, Table S2).

Overall, our results at the protein level indicate no significant permeability barrier damage in HS-NL skin, while HS-L interfollicular epidermis can be characterized by some; however, there were inconsistent changes, as some barrier molecules were downregulated while others were present in decreased levels.

### 3.3. Functional Investigation of the Permeability Barrier

To investigate the function of the permeability barrier in HS, TEWL was measured on the lesional and non-lesional areas in HS patients (n = 16) and compared to TEWL in healthy volunteers (n = 20) measured in the AGR (axillary) regions. Measurements were performed in triplicate, resulting in 48 measurements in each investigated region. The TEWL measurements in HS-L (58.48 ± 20.26 g/m^2^ h) and HS-NL (41.5 ± 20.15 g/m^2^ h) were not significantly different from TEWL in AGR skin (49.61 ± 20.81 g/m^2^ h), indicating no detectable barrier dysfunction in HS (Figure 1).

### 3.4. Confocal Microscopy

As our results at the mRNA and protein levels did not match (mRNA levels for several molecules decreased with no resulting changes in protein levels), we aimed to examine the organization of cell junctions by confocal microscopy.

**Figure 3:**
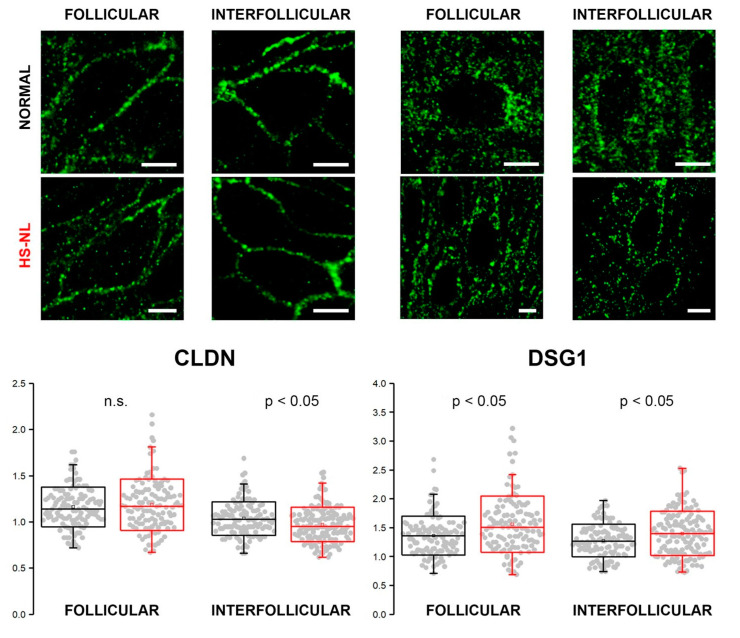
Representative images for immunofluorescent staining against CLDN (green; first and second columns from the left) and DSG1 (green; third and fourth column from the left) in the epidermis of normal and HS-NL (follicular and interfollicular) skin. Scale bar on all images: 5 µm. *Y*-axis contains inter-puncta distances in micrometers on the box-plots. n.s., not significant.

Immunofluorescent staining against CLDN (representing TJ structures) and DSG1 (representing (corneo)desmosomes) was performed to assess their expression in the epidermis of normal and HS-NL skin types (Figure 3). HS-L skin samples were not involved in these experiments due to the prominently damaged hair follicles.

We found similar and well-organized expression patterns of CLDN1 in the follicular and interfollicular epidemis of both HS-NL and AGR samples. The mean of the interpuncta distances in the interfollicular area was significantly smaller in HS-NL compared with AGR, while distances in follicular epidemis were similar in the two sample groups; however, the distribution of the distances largely overlapped (Figure 3).

DSG1 expression along the cell membrane was less organized than that of CLDN in all the four samples, and its staining pattern was similar in both of the conditions. The mean of interpuncta distances showed a significant elevation in HS-NL skin in both follicular and interfollicular regions, and the dispersion of the data point (i.e., variability of the interpuncta distances) was also larger in HS-NL skin both in the follicular and interfollicular regions (Figure 3).

In summary, the staining patterns of TJs and corneodesmosomes were similar in HS-NL and AGR samples, and the differences related to the distances of junction structures were very small and showed opposite directions (FC_HS-NLvs AGR_ = −1.08 and 1.1, respectively) confirming our previous functional (TEWL) and descriptive (IHC) findings.

## 4. Discussion

One of the many skin functions is to form and maintain a permeability barrier, providing the primary line of defence against various exogenous physical, biological and chemical stressors. At the same time, the permeability barrier prevents loss of endogenous water. The SC layer and the TJ network located in the stratum granulosum are mainly responsible for the permeability barrier function of the skin [11,12,13].

In several chronic inflammatory skin diseases, such as atopic dermatitis (AD) and probably papulopustular rosacea (PPR), permeability barrier damage is thought to trigger the primary steps of the disease through the activation of KCs [14,15,16,17,18,19,20,21,22]. In AD, both genetic and acquired barrier damage were proven to be able to activate KCs, and cytokine production (TSLP, IL-33, IL-25) by KCs initiates DC activation and, consequently, type 2 adaptive immune response and inflammation [14,19]. Recently a very similar permeability barrier impairment of facial skin was demonstrated in PPR, and the role of this barrier alteration was indicated in the initiation of the disease, although this has not been proven [21]. In HS, the role of KCs as primary immune activators has been suggested, but the role of barrier damage in KC activation has not been investigated thoroughly [4,5]. Therefore, we explored the characteristics of the permeability barrier in HS-L and HS-NL skin samples and compared them with healthy AGR skin. We aimed to investigate the SC and TJ, as the two major barrier elements of the permeability barrier. Molecules related to cornified envelope formation, corneocyte desquamation, intercellular lipid lamellae formation and (corneo)desmosome organization, along with TJ molecules, and barrier alarmins were analyzed at the mRNA and protein levels.

According to our findings, in HS-NL skin, neither the mRNA nor the protein expressions of the investigated molecules were significantly different from normal skin. In HS-L skin, we detected significantly different mRNA levels for 11 molecules compared to AGR skin; however, at the more relevant protein levels, these differences could not be strengthened. HS-L showed only slight, bidirectional alterations at the protein level (KRT1 and KLK5 decreased, KLK7, KRT6 and DSG1 increased). Among them, only KRT6 exhibited high fold changes in the same direction both at the mRNA and the protein level with significant increase in HS-L. The marked expression of KRT6 in HS has been reported by other research groups [4,23]. Since KRT6 is a marker of highly activated and proliferative KCs under pathological conditions rather than a barrier-forming molecule [24,25], our finding indicates abnormal KC proliferation/inflammation rather than a barrier defect.

Our functional and confocal microscopy investigations strengthened our immunohistochemical results, since severe barrier abnormalities were absent in both HS-L and HS-NL samples; the TEWL and confocal microscopy results indicated no significant damage in the function of the permeability barrier or the expression of junction structures.

To date, only a few studies investigated the permeability barrier in HS. These articles did not apply simultaneous RNA- and protein-based investigations or confocal methods and did not cover all main barrier elements [23,26,27]. Furthermore, previous studies included only lesional skin and not non-lesional skin. In HS, the healthy-looking non-lesional skin, which represents an intermediate stage between healthy and lesional skin of patients, is often used to detect early events (early mediators and cellular components) in the pathogenesis of the disease [5,28,29,30]. Therefore, HS-NL skin samples were included in our investigation.

Among the available studies examining barrier molecules, the CE structural components, FLG and LOR, were investigated by Navrazhina et al., and their results were similar to ours; no significant differences between the HS-L and control groups were detected. Of note, protein levels were not quantified in this publication [26]. The results of the two publications that investigated the expression levels of desmosome-composing molecules in HS were in agreement with our study, as DSG1 was increased in inflamed HS skin. However, they did not examine DSG1 mRNA levels, and no HS-NL samples were assessed [23,27]. To the best of our knowledge, changes in TJ molecules in HS have not been previously investigated.

In summary, our results suggest that the permeability barrier is not significantly affected in HS, and barrier damage does not play a prominent role in its initiation. Since HS is a follicular disease, our investigations at the protein level (by IHC and confocal microscopy) were fulfilled both in the interfollicular and the follicular epidermis, and none of them performed severe barrier damage. Our conclusion based on these results is that probably not barrier damage, but other triggers, such as mutations, mechanical stress, hormonal changes, altered production or pH of sweat and dysregulated microbiota may activate genetically sensitive KCs and initiate innate and subsequent adaptive immune inflammation, resulting in the clinical development of HS [31,32,33].

Limitation of the study: HS is a follicular disease, but the follicles in lesional HS are damaged to such an extent that only interfollicular epidermis could be examined in that sample at the protein level. Another limitation of the study is the small sample size.

## Data Availability

The data that support the findings of this study are available from the corresponding author upon reasonable request.

## Supplementary Materials

The following supporting information can be downloaded at: https://www.mdpi.com/article/10.3390/biomedicines11010127/s1, Table S1: Characteristics of skin samples from lesional and non-lesional areas of hidradenitis suppurativa (HS) patients and apocrine gland-rich (AGR) skin regions of healthy individuals;. Table S2: Comparison of permeability barrier components’ expression in healthy AGR skin and non-lesional and lesional HS by RT-qPCR and IHC.
