# Supplementary material for: New Data on the Features of Skin Barrier in Hidradenitis Suppurativa
Source: Biomedicines. 2023 Jan 4;11(1):127. doi: 10.3390/biomedicines11010127 (PMC9855647; doi:10.3390/biomedicines11010127)
Supplement: Supplementary file 1 [file biomedicines-11-00127-s001.zip › biomedicines-2116280-supplementary.pdf]

Table S1. Characteristics of skin samples from lesional and non-lesional areas of hidradenitis suppurativa (HS) patients and apocrine gland-rich (AGR) skin regions of healthy individuals

Hidradenitis suppurativa patients (n = 10)			
**HS individuals**	**Sex**	**Age**	**Localization**
HS1	M	29	axilla
HS2	M	25	axilla
HS3	M	61	axilla
HS4	M	35	axilla
HS5	M	24	axilla
HS6	F	30	axilla
HS7	M	20	axilla
HS8	F	51	axilla
HS9	F	31	axilla
HS10	F	43	axilla
Mean age ± SD		34.9 ± 13.0	

Healthy individuals (n = 10)			
**Healthy individuals**	**Sex**	**Age**	**Localization**
AGR1	F	60	axilla
AGR2	M	31	axilla
AGR3	F	38	axilla
AGR4	F	60	axilla
AGR5	M	19	axilla
AGR6	M	30	axilla
AGR7	F	55	axilla
AGR8	F	57	axilla
AGR9	F	61	axilla
AGR10	F	57	axilla
Mean age ± SD		46.8 ± 15.7	

Abbreviations: AGR, apocrine gland–rich; F, female; HS, hidradenitis suppurativa; M, male; SD, standard deviation

Table S2. Comparison of permeability barrier components' expression in healthy AGR skin and non-lesional and lesional HS by RT-qPCR and IHC

	**Non-lesional HS vs healthy AGR skin**				**Lesional HS vs Non-lesional HS skin**				**Lesional HS vs healthy AGR skin**			
**Variable**	**qRT-PCR**		**Epidermal IHC**		**qRT-PCR**		**Epidermal IHC**		**qRT-PCR**		**Epidermal IHC**	
	**P-value**	**FC**	**P-value**	**FC**	**P-value**	**FC**	**P-value**	**FC**	**P-value**	**FC**	**P-value**	**FC**
**Cornified envelope formation**												
FLG	0.3488	2.03	0.0636	2.24	0.0976	-2.39	>0.9999	-1.15	>0.9999	-1.18	0.4094	1.95
KRT1	>0.9999	-1.12	0.2751	-1.19	**0.0167**	**-3.04**	0.3755	-1.19	**0.0031**	**-3.34**	**0.0257**	**-1.42**
KRT10	0.8386	-1.10	ND	ND	**0.0044**	**-2.83**	ND	ND	**0.0015**	**-3.12**	ND	ND
LOR	0.2361	1.69	>0.9999	-1.16	**0.0103**	**-4.74**	0.0581	1.69	0.3064	-2.79	0.0876	1.46
TGM5	0.7368	1.15	>0.9999	-1.02	**0.0013**	**-3.38**	>0.9999	-1.02	**0.0103**	**-2.93**	>0.9999	-1.05
**Corneocyte desquamation**												
KLK5	0.5618	1.22	>0.9999	-1.09	**0.028**	**-1.94**	0.2582	-1.14	0.2212	-1.59	**0.0318**	**-1.26**
KLK7	0.214	1.37	0.0937	1.32	**0.0214**	**-1.85**	**0.0199**	**1.33**	0.4876	-1.35	**<0.0001**	**1.76**
KLK14	>0.9999	-1.49	ND	ND	0.9023	-1.03	ND	ND	0.2158	-1.53	ND	ND
**Desmosome formation**												
CDSN	>0.9999	1.28	0.7156	-1.11	**0.0092**	**-3.62**	0.937	-1.04	**0.026**	**-2.82**	0.8984	1.06
DSC1	0.7469	-1.16	ND	ND	**0.0063**	**-3.48**	ND	ND	**0.0016**	**-4.05**	ND	ND
DSG1	0.7986	-1.13	0.8672	1.16	**0.0052**	**-2.95**	0.0783	1.63	**0.0017**	**-3.33**	**0.0277**	**1.89**
PKP1	0.5841	-1.23	ND	ND	0.1259	-2.30	ND	ND	**0.0032**	**-2.98**	ND	ND
**Tight junction formation**												
CDH1	0.8401	1.09	ND	ND	**0.0017**	**-2.06**	ND	ND	**0.0118**	**-1.9**	ND	ND
CLDN1	0.6851	-1.29	0.9061	1.15	**0.0167**	**-3.83**	0.3623	-1.29	**0.0004**	**-4.97**	>0.9999	-1.12
OCLN	>0.9999	-1.06	0.064	-1.46	0.2408	-1.65	0.7466	1.14	0.2809	-1.76	0.2415	-1.28
**Barrier alarmin**												
KRT6A	0.4431	2.29	0.4928	14.43	0.9842	3.22	**0.0201**	**15.51**	**0.0453**	**7.37**	**0.0001**	**223.71**
KRT16	0.0561	4.61	ND	ND	>0.9999	1.78	ND	ND	**0.0295**	**8.22**	ND	ND

Abbreviations: AGR, apocrine gland–rich; CDSN, corneodesmosin; CLDN, claudin; DSG1, desmoglein 1; DSC1, desmocollin; FC, fold change; FLG, filaggrin; HS, hidradenitis suppurativa; IHC, immunohistochemistry; KLK, kallikrein-related peptidase; KRT, keratin; LOR, loricrin; ND, not determined; OCLN, occludin; PKP1, plakophilin; qRT-PCR, quantitative real-time PCR; TGM, transglutaminase.

Statistical analyses between protein and mRNA levels were determined by one-way analysis of variance followed by Sidak's post hoc test in case of normal data distribution or Kruskal-Wallis test followed by Dunn's post hoc test when data distribution was not normal. Bold type represent data with significant differences.
